# Health Inequalities: action starts with awareness

**DOI:** 10.1016/j.ebiom.2021.103376

**Published:** 2021-04-29

In July, 2020, in recognition of the *Lancet* pledge to advance racial equality, *EBioMedicine* committed to contributing to increase equality and diversity in science. Approaching this 1 year anniversary and in honour of minority health month, which is celebrated in the USA throughout April, we reflect on the progress made and look towards future efforts.

Issues of health equality that are more commonly associated with clinics and health policy might be overlooked when considering more basic aspects of biomedical research. Nevertheless, it is evident that inequalities in health and science arise from very early stages, and this happens both at a structural and at an experimental level.

Structural inequities in biomedical research might affect the progression and the opportunities of the researchers themselves, especially at the beginning of their career development. We need to ensure that voices of researchers belonging to underrepresented minorities and countries are heard and amplified, as too many times highly relevant efforts and initiatives are undermined by insufficient resources and visibility. Motivated by this mission, we reached out to researchers around the world and commissioned a series of letters focused on inequalities in paediatric health. Researchers who are experts in different areas, from mental health to pulmonary infectious diseases, to nutrition and oncology, shared views on their personal work experience, vision, and challenges. The series highlights how researchers at different levels of their career are working in difficult contexts to improve scientific knowledge and the health of their patients. While the project is still ongoing, some letters are already published in *EBioMedicine* and are also available in the *Lancet* hub for racial equality (https://www.thelancet.com/racial-equality).

Among the authors who contributed, Dr Rosemary Musesengwa (University of Oxford, Oxford, United Kingdom), who studies the ethics of digital mental health care for young people in Africa, highlighted that the challenges she encounters through her own work and via collaboration with a young-people led network (Ethics of Digital Innovation in Mental Health of Young People in Africa) were also issues she experienced first-hand as a Black woman. Dr Tufaria Mussá (Eduardo Mondlane University, Maputo, Mozambique), who studies paediatric infectious respiratory diseases, highlighted how investments and global collaborations should aim to strengthen both health-care systems and research projects, and emphasised the need to have local researchers involved at the very early stages of research proposals so that they can bring specific insights to the discussion. A resonating theme, spanning through topics and countries, was the importance of advocacy and appropriate training. These factors represent fundamental challenges for researchers, such as Dr Peter Rohloff (Maya Health Alliance, Chimaltenango, Guatemala), who is tackling chronic paediatric undernutrition in Guatemala, and Dr Fatima Mir (The Aga Khan University, Karachi, Pakistan), who is working to formalise the care of children with bronchiectasis in Pakistan. The creation of local networks of scientists and doctors is also important, and has been described as a game changer in paediatric oncology both by Dr Mhamed Harif, working at Centre Hospitalier Universitaire Tanger Tétouan Al Hoceima, Casablanca, Morocco, and Dr Sameer Bakhshi from All India Institute of Medical Sciences, New Delhi, India. The difficulties brought by the pandemic were also discussed, as Dr Toyin Togun (London School of Hygiene & Tropical Medicine, London, United Kingdom), who works between London and The Gambia to find better diagnostics for childhood tuberculosis, reflected on the effects exerted by COVID-19 in access to routine diagnosis and treatment services in high burden settings.

*EBioMedicine* believes it is of great importance to highlight and discuss these themes, and we hope to continue serving as a platform for such conversations, looking forward to the experiences of other translational researchers around the globe.

In addition to this, it is important to highlight that health inequalities can also occur at the experimental level. This problem is not limited to clinical research but can initiate in basic and translational biomedical experiments. Potential issues might originate from relying on human samples or datasets from restricted and non-diverse populations, decreasing generalisability, from doing experiments in preclinical models only on a single-sex, or by generating preliminary data from only a small number of cell lines. All these issues, which stem from inappropriate research designs and methodologies, are the first events in a chain generating and amplifying unbalances and bias in health. This bias can lead to underrepresentation of minorities and could ultimately be harmful to their health. A more self-conscious and inclusive approach is needed across the spectrum of translational medicine so that information that feeds subsequent clinical applications can be as comprehensive as possible. Only by ensuring that inclusion encompasses all aspects of research might we hope for a sustainable and real change. To this end, we are currently commissioning a series of review articles that reflect on and analyse these themes in a variety of translationally relevant topics.

Finally, we invite and encourage submissions that address issues of inequalities and underrepresentation in translational research, or that consider minorities, gender, and inclusive representation in their methods or sampling techniques. At *EBioMedicine,* we believe that raising awareness, inside and outside the journal, is the first step towards active change, and we intend to maintain and expand the pledge made last July. We aim to promote equitable research systems, from bench to bedside, that truly benefit everyone.

*EBioMedicine*

